# Kurarinone Inhibits HCoV-OC43 Infection by Impairing the Virus-Induced Autophagic Flux in MRC-5 Human Lung Cells

**DOI:** 10.3390/jcm9072230

**Published:** 2020-07-14

**Authors:** Jung Sun Min, Dong Eon Kim, Young-Hee Jin, Sunoh Kwon

**Affiliations:** 1 Herbal Medicine Research Division, Korea Institute of Oriental Medicine, Daejeon 34054, Korea; jsmin1019@kiom.re.kr (J.S.M.); ehddjs0@kiom.re.kr (D.E.K.); 2 Center for Convergent Research of Emerging Virus Infection, Korea Research Institute of Chemical Technology, Daejeon 34114, Korea; 3 KM Application Center, Korea Institute of Oriental Medicine, Daegu 41062, Korea

**Keywords:** kurarinone, coronavirus, HCoV-OC43, autophagy, infection, MRC-5 cell, LC3, p62/SQSTM1 protein

## Abstract

Kurarinone is a prenylated flavonone isolated from the roots of *Sophora flavescens*. Among its known functions, kurarinone has both anti-apoptotic and anti-inflammatory properties. Coronaviruses (CoVs), including HCoV-OC43, SARS-CoV, MERS-CoV, and SARS-CoV-2, are the causative agents of respiratory virus infections that range in severity from the common cold to severe pneumonia. There are currently no effective treatments for coronavirus-associated diseases. In this report, we examined the anti-viral impact of kurarinone against infection with the human coronavirus, HCoV-OC43. We found that kurarinone inhibited HCoV-OC43 infection in human lung fibroblast MRC-5 cells in a dose-dependent manner with an IC_50_ of 3.458 ± 0.101 µM. Kurarinone inhibited the virus-induced cytopathic effect, as well as extracellular and intracellular viral RNA and viral protein expression. Time-of-addition experiments suggested that kurarinone acted at an early stage of virus infection. Finally, we found that HCoV-OC43 infection increased the autophagic flux in MRC-5 cells; kurarinone inhibited viral replication via its capacity to impair the virus-induced autophagic flux. As such, we suggest that kurarinone may be a useful therapeutic for the treatment of diseases associated with coronavirus infection.

## 1. Introduction

Infection with human coronavirus HCoV-OC43 was first described in the 1960s; this virus is a representative and prototype of the virus family *Coronaviridae*. HCoV-OC43 is typically associated with mild respiratory tract infections. By contrast, the emergence of Severe Acute Respiratory Syndrome–associated coronavirus (SARS-CoV) in 2002 and the Middle East respiratory syndrome coronavirus (MERS-CoV) in 2012 proved that coronavirus pathogens can initiate cross-species infections and create significant healthcare risks [1]. Indeed, the novel SARS-CoV-2 pathogen has generated a global pandemic severe respiratory infection known as Coronavirus Disease 2019 (COVID-19). Although the antiviral agent, remdesivir, was recently authorized for emergency use for treatment for severely ill COVID-19 patients [2], the clinical utility and safety of remdesivir remains under investigation [3,4].

Viruses utilize various cellular processes to promote intracellular replication. Cellular autophagy is a particularly important feature promoting viral replication due to the fact that vesicle formation is critical to formation of autophagosomes and for transport of viral components [5]. The influenza A virus (IAV) [6], human immunodeficiency virus (HIV) [7,8], zikavirus (ZIKV) [9], and herpes simplex virus (HSV) [10] all use autophagy to promote viral replication and virion production [11]. Likewise, the hepatitis C virus (HCV) induces autophagy and can escape autophagic destruction [12] and treatment with an autophagy inhibitor-inhibited replication of the influenza A virus H3N2 [13]. Conversely, host cells also use autophagy as a means to inhibit viral replication to eliminate viral particles and to stimulate the immune response in order to prevent virus-induced disease. Autophagic processes can stimulate production of interferon and thereby promote innate immune signaling that ultimately results in degradation of the viral RNA genome and viral proteins (i.e., virophagy) [12,14,15]. The highly pathogenic avian influenza virus H5N1, Coxsackievirus B3, and HSV-1 were all reported to include mechanisms that facilitate escape from the autophagic degradation [12,16,17]. In the case of coronaviruses, SARS-CoV and the murine hepatitis virus (MHV) were reported to induce the formation of double-membrane-bound replication complexes that served to enhance viral replication [18]. Similarly, autophagic processes were critical features supporting the replication of the transmissible gastroenteritis virus (TGEV); furthermore, virus infection resulted in an increase in the autophagic flux [19].

Kurarinone is a prenylated flavonone isolated from the roots of the Asian shrub, *Sophora flavescens*; it is used as an analgesic in traditional Asian medicine. Kurarinone has been identified as an agent capable of inducing cell death by the activation of pro-apoptotic proteins and via the caspase-dependent pathway [20]; kurarinone also sensitizes TRAIL-induced tumor cell apoptosis via suppression of NF-κB-dependent cFLIP expression [21]. In other studies, kurarinone inhibited the development of chronic inflammatory dermatitis via suppression of CD4^+^ T cell differentiation [22]; combined administration of kurarinone and IFNα-1b promotes a positive response in patients with chronic hepatitis B [23]. Interestingly, kurarinone was shown to induce autophagic cell death via activation of autophagy-related proteins in human hepato-carcinoma cells [20]. However, to the best of our knowledge, there are no experimental reports directed at elucidating the antiviral impact of kurarinone.

In this study, we demonstrated that kurarinone inhibited HCoV-OC43 infection by interfering with virus-induced autophagy in the human lung cell MRC-5 line. As such, this study features a therapeutic approach to inhibit coronavirus infection by targeting virus-induced autophagy. Our work suggests that kurarinone may be useful as a novel therapeutic drug for the treatment of coronavirus disease.

## 2. Experimental Section

### 2.1. Preparation of Compounds

Kurarinone (PubChem CID: 10812923) was purchased from ChemFace (Wuhan, China), and remdesivir (PubChem CID: 121304016) was purchased from LALPharm Co., Ltd. (Beijing, China). Compounds were dissolved in dimethyl sulfoxide (DMSO), and stored as 20 mM stock solutions at −80°C. The stock solutions were diluted in serum-free culture medium prior to use. The final concentration of DMSO did not exceed 0.05%.

### 2.2. Cells and Virus Infection

The MRC-5 cell line was obtained from American Type Culture Collection (ATCC, Manassas, VA, USA). MRC-5 cells were cultured in Modified Eagle’s medium (MEM; Corning Incorporated, Corning, NY, USA) containing 10% fetal bovine serum (FBS; Gibco, Carlsbad, CA, USA) and 1% penicillin/streptomycin at 37 °C in 5% CO_2_. Human coronavirus-OC43 (HCoV-OC43) was obtained from ATCC and propagated and titrated, as previously described [24]. The titer of the purified HCoV-OC43 was 10^6.5^ TCID_50_ units (median tissue culture infectious dose)/100 µL. MRC-5 cells were seeded in 96-well plates at 5 × 10^3^ cells/well, infected with HCoV-OC43 (10^3.5^ TCID_50_/100 µL) and then incubated for 4 days at 33 °C.

### 2.3. MTS Assay

Cell viability was determined using the colorimetric 3-(4,5-dimethylthiazol-2-yl)-5-(3-carboxymethoxyphenyl)-2-(4-sulfophenyl)-2H-tetrazolium (MTS) assay (Promega Corporation, Madison, WI, USA) according to the manufacturer’s instructions. Absorbance was detected at 490 nm using a GloMax Microplate Reader (Promega).

### 2.4. Quantification of HCoV-OC43 RNA Copy Number

The viral RNA in the culture supernatants was isolated using a viral RNA purification QiaAMP kit (Qiagen, Hilden, Germany). Viral RNA in cell lysates were isolated using an RNeasy RNA purification kit (Qiagen) according to the manufacturer’s instructions. Quantitative reverse transcription PCR (qRT-PCR) was performed using the One Step SYBR^®^ PrimeScriptTM RT-PCR Kit (Takara Bio Inc., Shiga, Japan) according to the manufacturer’s protocol using primer pairs to amplify the HCoV-OC43 Nucleoprotein (NP) gene that included a sense primer, 5′-AGCAACCAGGCTGATGTCAATACC-3′, and an antisense primer, 5′-AGCAGACCTTCCTGAGCCTTCAAT-3′. The copy number was calculated using a standard curve of known concentrations of HCoV-OC43 RNA.

### 2.5. Western Blot Assay

MRC-5 cells were seeded in 24-well plates. Cells were harvested and lysed in Glo Lysis buffer (Promega). Proteins were separated on the SDS-PAGE gel and transferred to a nitrocellulose filter membrane (Bio-Rad Laboratories, Hercules, CA, USA). The membranes were blocked with 5% skim milk in tris-buffered saline with 0.5% Tween (TBST) for 30 min at room temperature (RT). The membranes were then rinsed with TBST and incubated with specific antibodies, including anti-viral-Spike protein (CusaBio Technology LLC, Houston, TX, USA), anti-LC3 protein (Abcam, Cambridge, United Kingdom), anti-p62/SQSTM1 protein (Abcam) or anti-β-actin (Cell Signaling Technology Inc., Danvers, MA, USA) at 4 °C overnight. Membranes were then incubated with horseradish peroxidase (HRP)-conjugated secondary antibodies (Abcam) for 1 h at RT and then developed with ECL solution (Thermo) using Chemidoc (Bio-Rad).

### 2.6. Quantification of Cytokine mRNA by qRT-PCR

Total RNA was isolated with the RNeasy^®^ Mini kit (Qiagen) according to the manufacturer’s instructions, and was used to synthesize complementary DNA (cDNA) using the One Step SYBR^®^ PrimeScript^TM^ RT-PCR Kit (Takara Bio) according to the manufacturer’s instructions. The following specific primers used for qRT-PCR were the following: for IFN-β1, sense primer 5′-ACCAACAAGTGTCTCCTCCA-3′ and antisense primer 5′-GTAGTGGAGAAGCACAACAGG-3; for β-Actin, sense primer 5′-GGAAATCGTGCGTGACATCA-3′ and antisense primer 5′-ATCTCCTGCTCGAAGTCCAG-3′.

### 2.7. Immunofluorescence Assay

MRC-5 cells were grown on coverslips; cells were fixed with 4% para-formaldehyde for 10 min and washed with phosphate-buffered saline (PBS). Cells were permeabilized with PBS containing 0.2% Triton X-100 for 10 min and blocked with 3% bovine serum albumin (BSA) for 30 min. After blocking, cells were incubated with anti-HCoV-OC43 S protein antibody (CusaBio) and/or anti-p62/SQSTM1 protein antibody (Abcam) at 4 °C overnight, followed by AlexaFluor555 goat-anti-rabbit IgG (ThermoFisher, Waltham, MA, USA) and/or AlexaFluor488 goat-anti-mouse IgG (ThermoFisher) at room temperature for 1 h. The labeled cells were mounted on slides with SlowFade Gold anti-fade reagent with DAPI (Invitrogen) and visualized by fluorescence microscopy (Olympus Corporation, Tokyo, Japan). Immunofluorescence data were quantified using ImageJ software (NIH).

### 2.8. Time-of-Addition Assay

MRC-5 cells (5 × 10^3^) were seeded in 96-well plates overnight. For the pretreatment assay, the cells were pre-treated with the compounds at concentrations indicated. After 24 h, the media were removed, and cells were washed and infected with HCoV-OC43 for 4 days at 33 °C. For the co-treatment and post-treatment assays, the compounds were added to the MRC-5 cell cultures during inoculation of the virus (co-treatment) or at 24 h after the virus was removed (post-treatment). At 4 dpi, the cell viability was determined using the MTS assay described above.

### 2.9. Statistical Analysis

The data were presented as the mean ± SEM. Statistical comparison by two-way analysis of variance (ANOVA) followed by Bonferroni’s multiple comparison’s test and non-linear regression analysis of IC_50_ and CC_50_ were conducted using GraphPad Prism^®^ Software V.6.05 for Windows (GraphPad Software Inc., San Diego, CA, USA). *P* values of less than 0.05 indicated statistical significance.

## 3. Results

### 3.1. Kurarinone Inhibited HCoV-OC43 Infection in MRC-5 Cells

**Figure 1:**
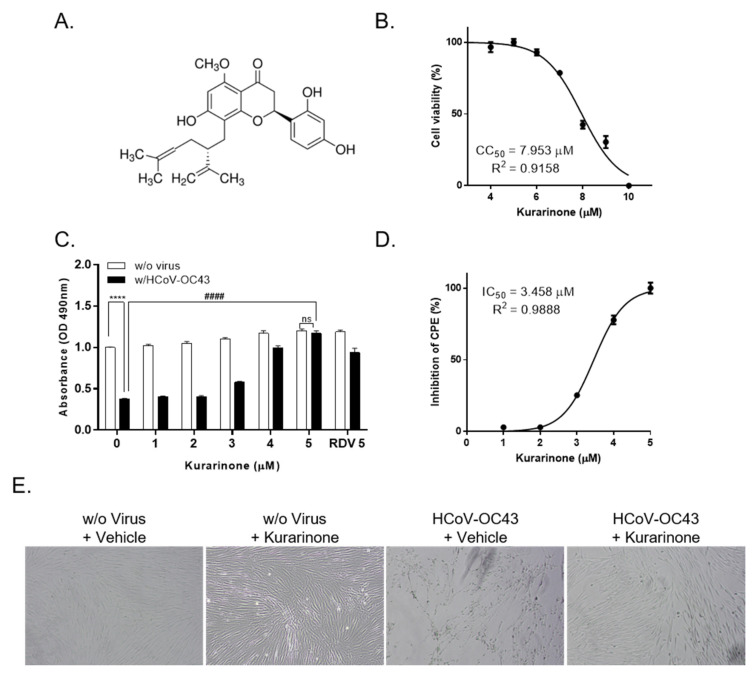
Chemical structure of kurarinone and antiviral activities in MRC-5 cells. (**A**) Chemical structure of kurarinone; (**B**) cytotoxicity associated with kurarinone. MRC-5 cells were incubated with increasing concentrations of kurarinone for 4 days; cell viability was measured by MTS assay (vehicle-treated cell as 100% of viability, 20% DMSO treated cell as 0% of viability). Cytotoxic concentration (CC_50_) of kurarinone was calculated after 4 days by nonlinear regression analysis; (**C**) Antiviral impact of kurarinone determined by degree of virus-induced cytopathic effect (CPE). MRC-5 cells were infected with HCoV-OC43 and incubated with various concentrations of kurarinone, or positive control, remdesivir (RDV) 5 µM for 4 days; cell viability was measured by MTS assay; (**D**) Inhibitory concentration (IC_50_) of kurarinone was calculated at 4 days post-infection (dpi) by nonlinear regression analysis. (vehicle-treated virus-infected cell as 0% of inhibition, vehicle-treated non-virus infected cell as 100% of inhibition); (**E**) Images of virus-infected MRC-5 cells at 4 dpi. Data were presented as means ± SEM of three independent experiments, and analyzed by two-way ANOVA with Bonferroni’s multiple comparisons test and nonlinear regression analysis. Virus effect, F(1, 36) = 1522; dose effect, F(5, 36) = 238.6; virus × dose interaction, F(5, 36) = 93.39; n.s., not significant; **** *p* < 0.0001; #### *p* < 0.0001.

Kurarinone is a flavonoid isolated from the roots of *Sophora flavescens* (Figure 1A). To examine its inhibitory activity with respect to HCoV-OC43 infection, we added kurarinone to uninfected or HCoV-OC43-infected MRC-5 cells for a period of 4 days. We determined the cytotoxic concentration (CC)_50_ of kurarinone for MRC-5 cells at 7.953 ± 0.148 µM by MTS assay (Figure 1B). We found that administration of kurarinone inhibited the virus-induced cytopathic effect (CPE) in a dose-dependent manner. As a positive control, 5 µM remdesivir was used (Figure 1C). An inhibitory concentration (IC)_50_ for kurarinone was calculated at 3.458 ± 0.101 µM by nonlinear regression analysis (Figure 1D). As such, we confirmed that kurarinone had antiviral activity against HCoV-OC43; we used 5 µM kurarinone for all further experiments. We then evaluated the impact of kurarinone on cell growth and morphology; we treated HCoV-OC43-infected MRC-5 cells with 5 µM kurarinone for 4 days and examined the morphology of cells by light microscopy. As shown in Figure 1E, HCoV-OC43 induced a clear cytopathic effect (CPE) that was detected in infected cells at 4 days post-infection (dpi); by contrast, cells treated with kurarinone had no virus-induced CPE and were indistinguishable from uninfected cells.

### 3.2. Kurarinone Inhibited HCoV-OC43 Replication and Viral Protein Expression in MRC-5 Cells

**Figure 2:**
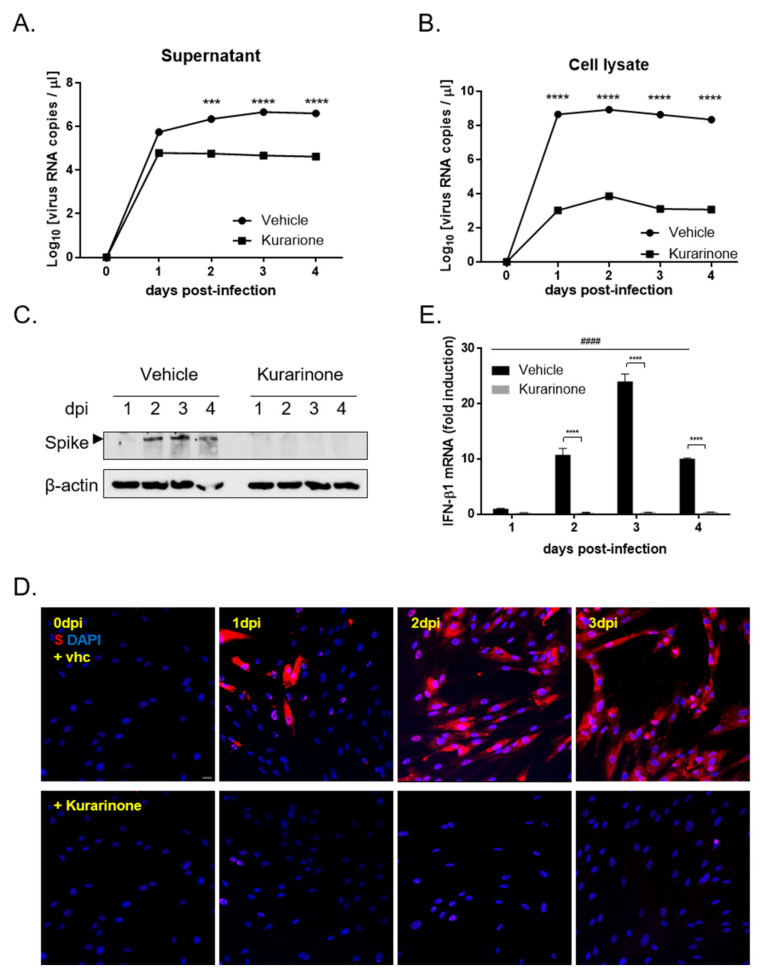
Detection of viral RNA and virus Spike protein in cell cultures treated with kurarinone. Viral RNA was purified from (**A**) culture supernatants or (**B**) cell lysates for quantification of HCoV-OC43 virus replication. RNA copy numbers were measured by qRT-PCR; (**C**) Western blot of the lysates of HCoV-OC43-infected MRC-5 cells treated with kurarinone or vehicle and evaluated at 1, 2, 3, and 4 dpi. The HCoV-OC43 Spike protein was detected and indicated by an arrowhead as shown; β-actin was used as a loading control. (**D**) Immunofluorescence analysis of HCoV-OC43-infected MRC-5 cells treated with vehicle (vhc) or kurarinone; cells were probed with an anti-viral Spike protein-specific antibody (red) and mounted with DAPI (blue) at 0, 1, 2, and 3 dpi. Scale bar is 50 µm. (**E**) Quantification of mRNA encoding interferon (IFN)-β1 by qRT-PCR in vehicle and kurarinone-treated MRC-5 cells; probes targeting the β-actin gene were used for data normalization. Data were presented as means ± SEM of three independent experiments, and analyzed by two-way ANOVA with Bonferroni’s multiple comparisons test. In (A), treatment effect, F(1, 8) = 218.7; dpi effect, F(4, 8) = 36.31; treatment × dpi interaction, F(4, 8) = 36.1; in (B), treatment effect, F(1, 8) = 2766; dpi effect, F(4, 8) = 338.5; treatment × dpi interaction, F(4, 8) = 338.5; *** *p* < 0.001, **** *p* < 0.0001 versus vehicle-treated group; in (E), treatment effect, F(1, 8) = 617.4; dpi effect, F(3, 8) = 112.7; treatment × dpi interaction, F(3, 8) = 112.0; **** *p* < 0.0001 versus virus-infected and vehicle-treated group, #### *p* < 0.0001 versus virus-infected vehicle-treated group at 1 dpi.

To examine the impact of kurarinone on virus replication, MRC-5 cells were infected with HCoV-OC43. Culture supernatants and cells pellet were harvested separately on days 1, 2, 3, and 4 post-infection; viral RNA levels were evaluated by qRT-PCR. As shown in Figure 2A, the level of HCoV-OC43 RNA in cell culture supernatant, which is the released viral RNA increased over time in cells treated with vehicle alone; the level of viral RNA in the supernatants of kurarinone-treated cells was significantly reduced. Consistent with the findings from cell culture supernatants, intracellular viral RNA was detected in MRC-5 lysates from vehicle-treated cells and at decreased levels in cells treated with kurarinone (Figure 2B).

We also evaluated HCoV-OC43 Spike protein expression in infected MRC-5 cells both with and without kurarinone treatment via Western blot analysis (Figure 2C). Viral Spike protein was first detected at 2 dpi in vehicle-treated cells; no S protein was detected in virus-infected and kurarinone-treated cells during the entire time period evaluated. Similarly, HCoV-OC43 Spike protein was detected in the cytoplasm of virus-infected cells examined by immunofluorescence at 1, 2, and 3 dpi; no Spike protein was detected in virus-infected and kurarinone-treated cells at this time point (Figure 2D).

To evaluate the impact of kurarinone treatment on the induction of the host antiviral response, we examined expression of the host antiviral gene, IFN-β1, in response to virus infection. IFN-β1 mRNA was induced prominently and time-dependently in MRC-5 cells during the 3 days after infection with HCoV-OC45, and then reduced at 4 dpi; by contrast, no IFN-β1 mRNA was detected in virus-infected cells that were treated with kurarinone (Figure 2E). These data suggested that the antiviral impact of kurarinone may not be related to its capacity to induce the host antiviral immune response.

### 3.3. Kurarinone Inhibits HCoV-OC43 Infection at the Early Stage of Virus Infection

**Figure 3:**
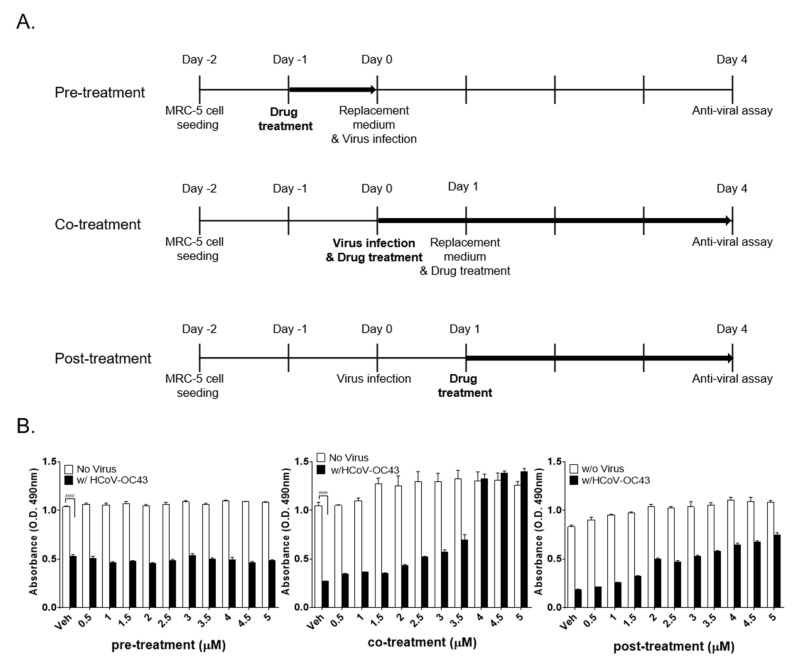
Time-of-addition assay to evaluate the impact of kurarinone. (**A**) Overall scheme for time-of-addition assay. The bold arrows denote the duration of kurarinone treatment. Kurarinone was added to MRC-5 cultures 24 h before infection, for 96 h during infection, or 24 h after virus infection; (**B**) Virus-induced cytopathic effect (CPE) was determined by MTS-based assay at 4 dpi. Data were presented as means ± SEM of three independent experiments, and analyzed by two-way ANOVA with Bonferroni’s multiple comparisons test; pre-treatment virus effect, F(1, 50) = 6494, *p* < 0.0001; dose effect, F(10, 50) = 1.695, *p* = 0.1081; virus × dose interaction, F(10, 50) = 2.478, *p* = 0.0170; co-treatment virus effect, F(1, 50) = 568.6, *p* < 0.0001; dose effect, F(10, 50) = 52.08, *p* < 0.0001; virus × dose interaction, F(10, 50) = 28.85, *p* < 0.0001; post-treatment virus effect, F(1, 50) = 3631, *p* < 0.0001; dose effect, F(10, 50) = 101.9, *p* < 0.0001; virus × dose interaction, F(10, 50) = 16.00, *p* < 0.0001; n.s., not significant; **** *p* < 0.0001 versus virus-infected vehicle-treated group.

In an effort to identify the stage of the HCoV-OC43 life cycle that is affected by the administration of kurarinone, we designed pre-treatment (kurarinone added for a period of 24 h prior to infection), co-treatment (kurarinone together with virus for the full 96 h), and post-treatment experiments (kurarinone added for 24 h after virus was introduced; Figure 3A). As shown in Figure 3B, when kurarinone was added to the cell culture before virus infection (pre-treatment), no protection against virus-induced CPE was observed. By contrast, the addition of kurarinone for a period of 24 h post-infection (post-treatment) resulted in up to 70% protection against virus-induced CPE, while co-treatment with kurarinone at a 5 µM concentration completely abolished virus-induced CPE (Figure 3B). These data suggested that the antiviral effect of kurarinone was focused on the early stages of virus infection; kurarinone was clearly most effective when added within 24 h of initial HCoV-OC43 infection.

### 3.4. Kurarinone Inhibits the HCoV-OC43 Infection by Modulating the Autophagy

**Figure 4:**
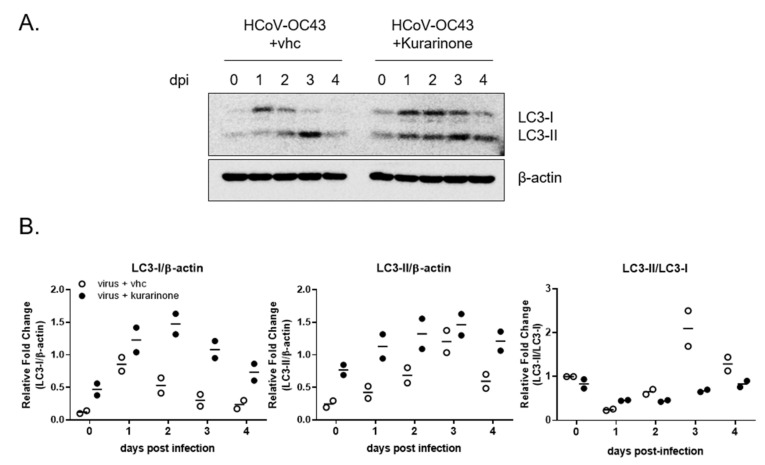
Autophagy induced by HCoV-OC43 infection was impaired by administration of kurarinone; (**A**) MRC-5 cells were infected with HCoV-OC43, treated with kurarinone or vehicle (vhc), and evaluated post-infection (dpi) on days 0, 1, 2, 3, and 4 by Western blot probed with anti-LC3 protein antibody; β-actin was used as the internal loading control. (**B**) The protein band intensities of LC3-I and LC3-II were quantified. Data were presented as means of two independent experiments.

Earlier reports indicate that infection with the MHV coronavirus-induced autophagy was required for effective virus replication [25]; kurarinone was also reported to modulate the expression of autophagy-associated protein [20]. As such, we proceeded to explore whether kurarinone was capable of modulating autophagy in HCoV-OC43-infected cells. To monitor autophagy, expression of LC3-I and LC3-II was examined in virus–infected cells by Western blot in cultures both with and without kurarinone (Figure 4A); the relative intensities of the protein bands are as shown in Figure 4B.

LC3-I expression increases at day 1 of HCoV-OC43 infection, and then falls thereafter, beginning on 2 dpi. Levels of phosphatidylethanolamine (PE)-conjugated LC3-I, LC3-II gradually increase until 3 dpi, and then decrease thereafter. The LC-3-I/II transversion (LC3-II/LC3-I ratio), an indicator of autophagy activity, increases steadily until 3 dpi and then decreases at day 4; these results suggest that autophagy increases for 3 dpi and that LC3-II undergoes autophagic degradation, beginning at 4 dpi [26]. Taken together, these data confirm that HCoV-OC43 induced cellular autophagy in infected MRC-5 cells; these findings are consistent with those reported previously for infection with other coronaviruses [27]. However, expression of both LC3-I and LC3-II proteins increased simultaneously for 2 dpi in response to administration of kurarinone to levels that were higher than those of either untreated infected cells or cells treated with vehicle alone. The level of LC3-I protein decreased, starting at 3 dpi; levels of LC3-II remained high for 3 dpi and were not degraded at 4 dpi, as were those detected in virus-infected cells and cells treated with vehicle alone. Finally, LC-3-I/II transversion was lower in virus-infected cells treated with kurarinone than in those of vehicle-treated and virus-infected cells alone; this was largely due to persistently high levels of LC3-I. Taken together, these results suggest that kurarinone has an impact on cellular autophagy induced by HCoV-OC43 infection.

**Figure 5:**
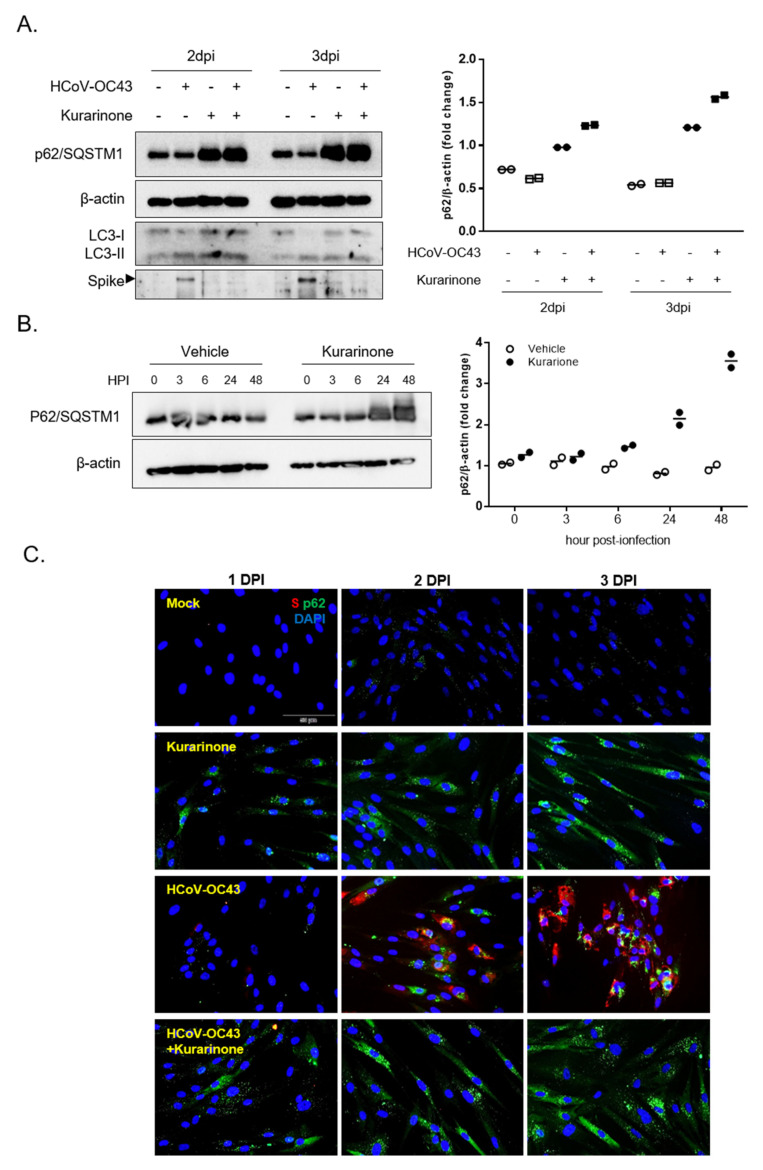
Expression of p62/SQSTM1 protein in response to administration of kurarinone; (**A**) HCoV-OC43-infected MRC-5 cells were treated with kurarinone or vehicle for 2 or 3 days; Western blot was performed with anti-p62/SQSTM1, -LC3, and -viral Spike antibodies at 2 or 3 dpi. (**B**) Virus-infected MRC-5 cells treated with kurarinone or vehicle alone were harvested at 3, 6, 24 or 48 h post-infection (hpi); cell lysates were analyzed by Western blot. Data were presented as means of two independent experiments. (**C**) Immunofluorescence analysis of HCoV-OC43-infected MRC-5 cells treated with vehicle (vhc) or kurarinone; cells were probed with an anti-viral Spike protein-specific antibody (red), p62/SQSTM1 protein-specific antibody (green) and DAPI (blue) at 1, 2, and 3 dpi. Scale bar is 50 µm.

Finally, we examined expression of p62/SQSTM1 protein, a recognized indicator of autophagic flux together with the expression of LC3 protein and viral Spike protein (Figure 5A). Expression p62/SQSTM1 protein was time-dependently reduced during virus infection (Supplementary Figure S1A); these results indicate that the autophagy flux was increased in response to virus infection. Interestingly, p62/SQSTM1 protein levels were increased at days 2 and 3 in those that were kurarinone-treated, including those both infected and uninfected by HCoV-OC43. Furthermore, we determined the time to kurarinone-mediated induction of p62/SQSTM1 at 3, 6, 24, and 48 hpi. Levels of p62/SQSTM1 protein increased from 6 h of treatment, reaching a maximum at 48 h and remaining sustained throughout the period of virus infection (Figure 5B). Moreover, the immunofluorescence assay showed that HCoV-OC43-infected and kurarinone-treated cells exhibited a higher level of p62/SQSTM1 protein than those of HCoV-OC43 infected cells at 1, 2, and 3 dpi (Figure 5C). Some cells showed the increased level of p62/SQSTM1 protein without Spike protein expression. It may be the transiently increased p62/SQSTM1 protein in cells where there is an increase in autophagy flux at initial stage of virus infection, before Spike protein is expressed. We also confirmed that autophagy inhibitors, NH_4_Cl, and Chloroquine induced the p62/SQSTM1 proteins, which were known to inhibit the coronavirus infection (Supplementary Figure S1B). As such, these data suggest that kurarinone can inhibit HCoV-OC43 infection by inducing expression of p62/SQSTM1 protein and thereby impairing the autophagic flux.

## 4. Discussion

In this study, we explored mechanisms underlying the anti-viral activity of kurarinone on coronavirus HCoV-OC43 infection in human lung MRC-5 cells. Kurarinone is a prenylated flavonone isolated from the roots of *S. flavescens* that has well-characterized pro-apoptotic and anti-inflammatory effects. However, to the best of our knowledge, there are no published reports documenting the antiviral activities of kurarinone, save for one clinical report suggested that the combination of kurarinone and IFNα-1b might be used to treat patients with chronic hepatitis B [23].

In our first set of experiments, we found that kurarinone treatment inhibited HCoV-OC43 infection in MRC-5 cells in a dose-dependent manner with an IC_50_ 3.458 ± 0.101 µM (Figure 1C,D). Kurarinone treatment resulted in diminished levels of extracellular and intracellular viral RNA and diminished expression of the virus Spike protein compared to the virus-infected and vehicle-treated controls (Figure 2). Pre-treatment with kurarinone prior to virus infection did not protect against virus-induced CPE. Kurarinone treatment at 24 h post-infection resulted in partial protection against virus-induced CPE; as such, our results suggest that kurarinone may have a primary antiviral effect at the early stages of infection (Figure 3). To determine whether kurarinone has the capacity to modulate cytokine expression in HCoV-OC43-infected cells, we examined the kinetics of the antiviral cytokine, IFN-β1. The mRNA encoding IFN-β1 was significantly upregulated by virus infection but could not be detected at all in virus-infected and kurarinone-treated cells (Figure 2E). These data indicated that the antiviral response by kurarinone was not related to the induction of this antiviral cytokine.

Autophagy is a metabolic process that involves the intracellular membrane transport pathway and promotes recycling of proteins and organelles. Autophagy is also a critical factor with respect to virus infection and virion propagation [6,7,8,9,10]. During the stage of viral entry, HCoV-OC43 entered the cells by 9-O-acetylated sialic acids-mediated endocytosis, which is the determinant of host and tissue tropism [28]. Some viruses induced the early wave of autophagy induction upon virus infection, which may have contributed to viral entry [29]. Moreover, the coronavirus required the autophagy process for the formation of double-membrane-bound MHV replication complexes, which can significantly enhance the efficiency of replication [25]. As reported in a previous publication, treatment with the autophagy inhibitor, NH_4_Cl, which alters lysosomal pH, limited the extent of HCoV-OC43 infection [29]. These results suggested that HCoV-OC43 infection induced autophagic processes, a finding that is consistent with observations associated with other coronavirus infections [18,28]. To verify the cellular status of autophagy and to monitor autophagic flux during HCoV-OC43 infection, we evaluated the expression of LC3 and p62/SQSTM1 proteins [19] (Figure 4 and Figure 5). In HCoV-OC43-infected cells, we detected autophagy up to and including 3 dpi, as documented by the LC-3-I/II transversion.

Interestingly, treatment with kurarinone resulted in increased expression of LC-3-I and LC-3-II for 2 dpi; the LC-3-I/II transversion detected in kurarinone-treated cells was lower than that identified in vehicle-treated cells due to the comparatively high levels of LC3-I expression. These results suggested that kurarinone might disturb the HCoV-OC43-induced autophagy process. Consistent with this hypothesis, levels of the autophagic degradation substrate, p62/SQSTM1, were decreased in HCoV-OC43 infected cells; these results suggest that HCoV-OC43 infection induced the autophagic flux. However, kurarinone treatment induced the accumulation of p62/SQSTM1 protein in HCoV-OC43-infected cells starting at 6 h post-infection; p62/SQSTM1 protein-bound ubiquitinated protein is incorporated and degraded in the autophagosome, which indicates an autophagic degradation [30]. During the early stages of HSV-1 infection, down-regulation of p62/SQSTM1 protein is important for viral gene expression; exogenous overexpression of p62/SQSTM1 protein decreased the viral load, suggesting that p62/SQSTM1 protein has an antiviral function [31,32]. Our data also suggested that the increased expression of p62/SQSTM1 protein induced by kurarinone may have an important role in inhibiting HCoV-OC43 infection in MRC-5 cells. Therefore, kurarinone could not block the viral entry, as indicated by no antiviral effect of 24 h pre-treatment. The induced p62/SQSTM1 protein and disturbed autophagy flux within 24 h post-infection were important for the antiviral effect of kurarinone, and the weakened antiviral effect of 24 h post-infection treatment suggests that the benefits of kurarinone treatment may be optimal at the initial infection stages.

In conclusion, our results indicate that kurarinone can inhibit the progression of coronavirus infection by interrupting virus-induced autophagy. Therefore, this study outlines a therapeutic approach that may be used to inhibit coronavirus infection by targeting the virus-induced autophagy. These data suggested that kurarinone may be considered to be the basis of a novel and useful prophylaxis for high-risk exposure groups and therapeutic regiment for the treatment of diseases associated with coronavirus infection at the initial infection stage. Although we found the therapeutic potentials of kurarinone against HCoV-OC43 infection, the proof of concept (POC) was executed only in vitro, and the selectivity index was only 2.29 folds, which may have acted as a hurdle of its application in the clinic. Therefore, pharmacokinetic study and an in vivo toxicity test should be executed to determine its biostability and biosafety, and in vivo POC should also be confirmed in further study.

## Supplementary Materials

The following are available online at https://www.mdpi.com/2077-0383/9/7/2230/s1, Figure S1: Expression of p62/SQSTM1 protein in response to administration of kurarinone and autophagy inhibitors (NH_4_Cl and Chloroquine).
