# Supplementary material for: Kurarinone Inhibits HCoV-OC43 Infection by Impairing the Virus-Induced Autophagic Flux in MRC-5 Human Lung Cells
Source: J Clin Med. 2020 Jul 14;9(7):2230. doi: 10.3390/jcm9072230 (PMC7408680; doi:10.3390/jcm9072230)
Supplement: Supplementary file 1 [file jcm-09-02230-s001.pdf]

# Supplementary Fig.1

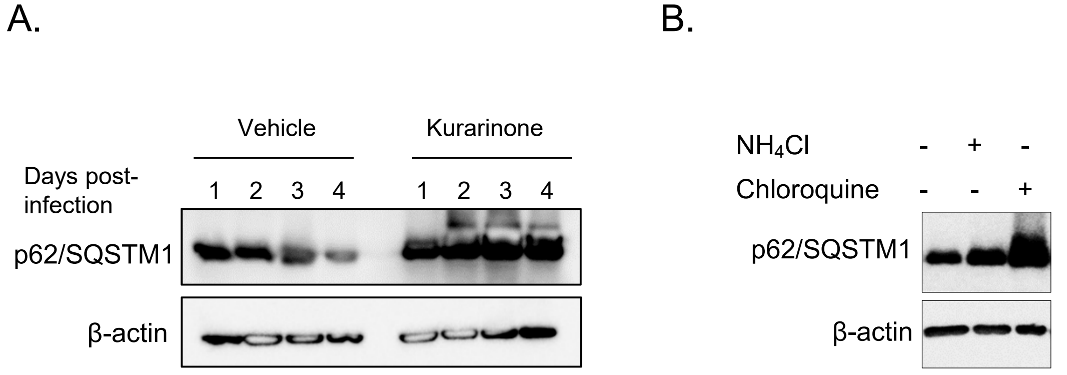


**Supplementary Figure 1**. Expression of p62/SQSTM1 protein in response to administration of kurarinone and autophagy inhibitor ($NH_4Cl$ and Chloroquine). (A) HCoV-OC43-infected MRC-5 cells were treated with kurarinone or vehicle for 4 days; Western blot was performed with anti-p62/SQSTM1. (B) MRC-5 cells treated with 5 mM $NH_4Cl$ or 10 μM Chloroquine for 2 days were harvested and cell lysates were analyzed by Western blot.
